# Selection on an antagonistic behavioral trait can drive rapid genital coevolution in the burying beetle, *Nicrophorus vespilloides*

**DOI:** 10.1111/evo.12938

**Published:** 2016-05-24

**Authors:** Paul E. Hopwood, Megan L. Head, Eleanor J. Jordan, Mauricio J. Carter, Emma Davey, Allen J. Moore, Nick J. Royle

**Affiliations:** ^1^ Centre for Ecology and Conservation, College of Life and Environmental Sciences, University of ExeterCornwall CampusPenrynTR10 9FEUnited Kingdom; ^2^ Division of Evolution, Ecology and Genetics, Research School of BiologyAustralian National UniversityActonACT0200Australia; ^3^ Centro Nacional del Medio Ambiente. Fundación de la Universidad de ChileAv. Larrain 9975La ReinaSantiagoChile; ^4^ Department of GeneticsUniversity of GeorgiaAthensGeorgia30602

**Keywords:** Artificial selection, burying beetle, genital morphology, repeated mating, sexually antagonistic coevolution, sexual conflict, sexual selection

## Abstract

Male and female genital morphology varies widely across many taxa, and even among populations. Disentangling potential sources of selection on genital morphology is problematic because each sex is predicted to respond to adaptations in the other due to reproductive conflicts of interest. To test how variation in this sexual conflict trait relates to variation in genital morphology we used our previously developed artificial selection lines for high and low repeated mating rates. We selected for high and low repeated mating rates using monogamous pairings to eliminate contemporaneous female choice and male–male competition. Male and female genital shape responded rapidly to selection on repeated mating rate. High and low mating rate lines diverged from control lines after only 10 generations of selection. We also detected significant patterns of male and female genital shape coevolution among selection regimes. We argue that because our selection lines differ in sexual conflict, these results support the hypothesis that sexually antagonistic coevolution can drive the rapid divergence of genital morphology. The greatest divergence in morphology corresponded with lines in which the resolution of sexual conflict over mating rate was biased in favor of male interests.

Genital morphology is often disproportionately diverse compared to other morphological traits even among closely related species (Eberhard 1985; Hosken and Stockley 2004; Arnqvist and Rowe 2005; Simmons 2014). Several evolutionary mechanisms have been hypothesized to account for genital divergence (Arnqvist 1998; Hosken and Stockley 2004; Eberhard 2010) but recent theoretical and empirical work supports sexual selection as the key driver of genital diversification. Cryptic female choice could drive genital evolution if female genital traits facilitate biasing of paternity toward “preferred” males (e.g., Briceño and Eberhard 2009). Alternatively, selection may act on male genital traits associated with competition for fertilization success (Arnqvist 1997). A well‐known example of the latter scenario is retrorse hairs on intromittent organs of male damselflies that remove rivals’ sperm from premated females’ sperm storage structures (Waage 1979). However, genital traits predominantly selected to benefit individuals of one sex are likely to have implications for individuals of the other sex due to intersexual conflicts of interest (Parker 1979; Kokko and Jennions 2014; Parker 2014). For example, in seed beetles male genital spines may reduce the chance of an individual male being dislodged during intromission thus enhancing his relative mating success. However, as a side effect the female genital tract suffers damage from matings (Rönn et al. 2007). This type of conflict generates the potential for selection for female defensive counter‐adaptations that mitigate costs, leading to sexually antagonistic coevolution (Arnqvist and Rowe 2005). Mating with males that are successful by virtue of adaptations that circumvent female defensive counter‐adaptations can still provide indirect benefits for females via their own successful sons (Kokko 2005; Kokko and Jennions 2014). Thus, reproductive fitness for each sex potentially involves conflict between the sexes, the extent of which might vary with regard to which sex is subjected to the strongest selection for counter‐responses (Holland and Rice 1998; Gavrilets et al. 2001; Hosken and Stockley 2004; Arnqvist and Rowe 2005; Kokko and Jennions 2014).

Quantitative genetic studies have demonstrated a genetic basis that could underlie patterns of genital coevolution as one sex responds to the adaptations of the other (Sasabe et al. 2010; Simmons and Garcia‐Gonzalez 2011; Evans et al. 2013). Furthermore, patterns of coevolution between male and female genital structures have recently been found among closely related species at the phylogenetic level (Yassin and Orgogozo 2013; Burns and Shultz 2015). Under sexually antagonistic coevolution the sex currently having the “upper hand” may change through time and different mechanisms of sexual selection may be acting on alternate traits in each sex during different copulatory phases (Kokko and Jennions 2014; Parker 2014). This makes establishing clear mechanisms of evolutionary cause and effect problematic even in the few experimental studies that have looked at patterns of genital coevolution between males and females (Evans et al. 2011; Simmons and Garcia‐Gonzalez 2011; Evans et al. 2013; Yassin and Orgogozo 2013). This is because the functional relationship between variation in genital morphology and fertilization success (were they known) are interdependent even though the interests of males and females are never perfectly aligned (Arnqvist 1997; Eberhard 2004; Arnqvist and Rowe 2005; Simmons 2014).

In this study, we test how sexual conflict might influence the evolution of male and female genitalia in *Nicrophorus vespilloides* using our existing artificial selection lines selected for either high, control, or low repeated mating rates. In these lines the effects of cryptic female choice were controlled by excluding the effects of mate choice and sperm competition. Using these lines we have previously shown that there is sexual conflict over repeated mating rate, with high repeated mating rates being more costly for females than low rates of repeated mating (Head et al. 2014). For males however, high repeated mating is beneficial as a paternity protection mechanism (Müller and Eggert 1989; House et al. 2008). Our selection lines represent two scenarios in which either one sex or the other appears to be favored (i.e., females suffering minimal harassment by males in low lines vs. females facing repeated mating attempts from persistent males in high lines). Our aims, by directly manipulating a conflict trait, were both to test whether male and female genital morphology would coevolve and also identify morphological structural variation upon which selection may act.

## Methods

### ORIGIN AND MAINTENANCE OF BURYING BEETLES

Our stock population of *N. vespilloides* was established from 90 males and 90 females collected from Devichoys Wood, Cornwall, UK (N50º11’47’’E5º7’23’’) in July 2010 (for a brief summary of burying beetles as a model system see Royle et al. 2013). Full details of stock maintenance are given in Head et al. (2012). Briefly, we maintained the stock by breeding 50–60 pairs per generation. Each generation males and females were randomly paired for breeding, while avoiding brother–sister and first cousin matings. Additionally, beetles never contributed more than one brood to the following generation. To breed, each pair of virgin male and female beetles were placed in individual breeding chambers (17 × 12 × 6 cm) with 2 cm of moist soil and a 15—25 g mouse carcass (Livefoods Direct Ltd., Sheffield, UK). Once larvae dispersed from the mouse carcass they were removed from the breeding chamber and placed in individual rearing containers (7 × 7 × 4cm). After eclosion, beetles were sexed and fed two decapitated mealworms twice a week until they reached sexual maturity (∼14 days posteclosion). All rearing was conducted in a constant temperature room at 21 ± 1ºC with a 16L:8D light regime.

### SELECTION REGIME

Full details of our artificial selection regime are given in Head et al. (2014). In brief, we established and maintained two replicates of each line and maintained all lines at the same population size (we always avoid brother–sister and first cousin combinations). In each of 10 generations of selection males and females were mated monogamously controlling for mating competition and mate choice in both sexes. Using geometric morphometric analysis we tested whether male and female genital shape evolved in response to selection on repeated mating rate and if so whether the change in male and female genital shape resulting from selection on repeated mating rate was correlated. Given that we used monogamous pairings to eliminate potential effects of cryptic female choice and sperm competition, changes in genital morphology that were correlated with selection on mating rate or coevolution of male and female genital morphology provides evidence that sexually antagonistic coevolution is capable of altering genital morphology. Our F0 generation was derived from randomly paired 107 males and females (avoiding brother–sister and first cousin matings) and mating rate was recorded (number of times mating occurred in 1 h), before being allowed to breed. Offspring from families with the top ∼30% (33 families) and the bottom ∼30% (34 families) values of parental mating rate were allocated to the High (H) and Low (L) mating regimes, respectively. The Control (C) lines (30 families) were derived from randomly selected pairs, independent of mating rate (i.e., drawn from the whole pool of 107 pairs). All larvae were kept from breeding attempts meaning that each of the three different regimes consisted of ∼800–1000 individuals.

In the F1 generation, we split each selection regime into two different replicates to create a total of six lines (i.e., H1, H2, C1, C2, L1, L2), which allows us to control for drift. The replicates were created by randomly allocating males and females to pairs, with half (82 pairs) randomly allocated to replicate one and the other half (82 pairs) allocated to replicate two within each selection regime. Once the replicates were set up the top (H lines), bottom (L lines) or a random selection of 35 families was chosen to contribute to the next generation (∼800–1000 individuals per line). In the subsequent, F2 generation, and beyond, mating rate was measured for 100 randomly paired males and females (avoiding brother–sister matings) in each of the six lines and the top (H lines), bottom (L lines), or random 20–25 families chosen (i.e., a population size of ∼400–500 individuals per line per generation). Beetles within these selection lines were bred and reared as outlined above for stock beetles.

### EXPERIMENTAL DESIGN

To investigate how selection on repeated mating rate influences the evolution of male and female genitalia we conducted geometric morphometric shape analysis of a sample of male and female beetles (16–20 beetles of each sex from each line) from the tenth generation of selection of each of the six selection lines described above. Genitalia were dissected from sexually mature, virgin male and female beetles that had been euthanized and stored in a –20^º^C freezer (∼6 months prior).

**Figure 1:**
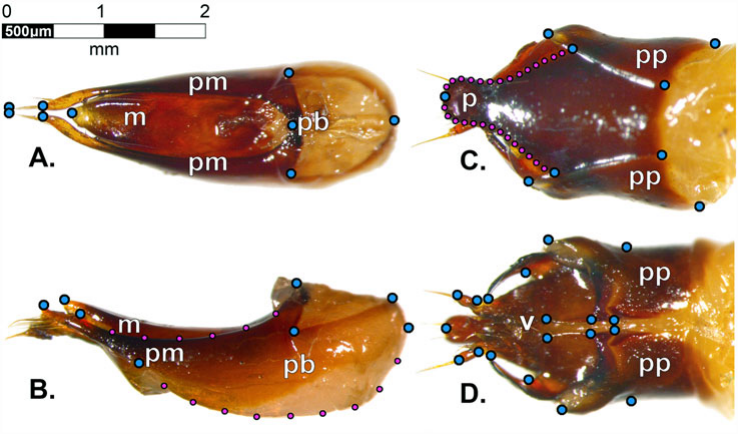
Micrographs of *N. vespilloides* genitalia showing positioning of fixed landmarks (blue‐large‐points) and semilandmarks (magenta‐small‐points): male (A: dorsal view and B: left lateral view) and female (C: dorsal view and D: ventral view). Lower case letters indicate genital structures: median lobe (*m*); parameres (*pm*); phallobase (*pb*); paraproct (*pp*); proctiger (*p*); vulva (*v*).

Prior to dissection beetles were removed from the freezer, allowed to defrost and their mass was recorded (to 0.001g, using an Ohaus, Explorer microbalance). Once beetles had thawed we dissected male and female genitalia. Dissections were performed on wax filled petri dishes with a pair of fine forceps and micro‐scissors under a dissecting microscope (Leica M125). For both males and females, the posterior abdominal segment (which houses the genitalia) was separated from the rest of the beetle. This was achieved by making an incision in the cuticle just above the required segment and cutting along the sides of the cuticle so that the final segment could gently be pulled out and placed in a clear petri dish. For males, the aedeagus was then removed by gently pulling away the tergites, pygidium, and remaining membranous tissue. The parameres and aedeagus were left intact, mounted onto a glass slide using petroleum jelly and photographed immediately. Care was taken to position genitalia in the same plane in all photos. The female genitalia were removed and mounted in a similar way. We photographed mounted male and female genitalia using a Leica M125 microscope with mounted camera that conveyed images to a PC. Digital images were processed using Image J. For males, we photographed the lateral and ventral view of the genitalia, while for females we photographed the dorsal and ventral view (Fig. 1).

### MORPHOMETRIC ANALYSIS

In order to quantify variation in the shape and size of the genitalia we used geometric morphometric analysis (Adams et al. 2004). Landmarks for all images were digitized (using software tpsDig version 2.12; 25) and are given in Figure 1. To conduct geometric morphometric analysis we followed the methods outlined in Zelditch et al. (2012) for images with bilateral symmetry and, when appropriate, semilandmarks (using software tpsRelw version 1.46; (Rohlf 2008)) and morphoJ software (http://www.flywings.org.uk/MorphoJ_page.htm).

Landmarks to be digitized were chosen based on their ease and reliability of placement while semilandmarks were used on curved structures with no insertion points. All dissections and photography were performed by one person (E. Jordan) blind with respect to the selection regime from which beetles came. Landmark digitization was similarly performed by one person (M. Head) blind to selection regime. Collecting data in this way was intended to minimize measurement error and prevent observer bias. Once the landmarks had been digitized and superimposed, we obtained relative warps (RW) from each of the images (using software tpsRelw version 1.46; (Rohlf 2008)). This program uses Procrustes methods to standardize each set of images to a common size, as well as center and align the landmarks so that differences in size and 2‐dimensional positioning of the genitalia do not contribute to shape differences between images. The tpsRelw software then calculates a consensus configuration from the standardized coordinates and compares each set of coordinates to the consensus configuration using thin‐plate spline analysis (Bookstein 1991). The method deforms each set of coordinates toward the consensus configuration, producing a unique set of energy values called “partial warps.” The principal components of these partial warps, called “relative warps,” summarize the major trends of shape variation in the set of images (Rohlf 1999). We conducted a single shape analysis for each image type. This means that individuals from different selection lines were all scored (for each image type) along the same axes of shape variation.

### DATA ANALYSIS

To investigate whether selection on repeated mating rate influenced the evolution of male and/or female genitalia we first conducted a discriminant function analysis (DFA) on the relative warps obtained from the geometric morphometric analyses detailed above. We conducted DFA for males and females separately. For each sex we included all relative warps that explained up to 99% of the shape variation in each of the two images for that sex. For females, this included relative warps 1–15 for the ventral view, and relative warps 1–12 for the dorsal view. For males, this included relative warps 1–15 of the lateral view and relative warps 1–7 of the dorsal view. Selection line was used as the grouping variable for both male and female analyses. Thus the first discriminant function gives a score representing the weighted linear combination of relative warps that best discriminates between selection lines, while the second discriminant function gives a score that best discriminates between selection lines based on the remaining shape variation described by the relative warps, and likewise for subsequent discriminant functions.

Using the discriminant function scores resulting from this analysis we then looked to see whether there were any consistent differences in male and female genital shape associated with selection regime. To do this, we conducted univariate nested ANOVA, for both males and females, on each of the five discriminate functions. In these analyses selection line was nested within selection regime as a random factor. We also conducted analyses using MCMCglmm that allows multivariate analysis with nested designs. This analysis (Tables S1.1 and S1.2) gave qualitatively similar results to our univariate analyses and so for ease of presentation and interpretation we present only the univariate analyses in this manuscript.

After determining whether male and female genitalia differed depending on selection regime we then looked to see if male and female genitalia had coevolved that is whether shape variation in male genitalia was correlated with shape variation in female genitalia. To do this, we performed bivariate correlations on line means of the first three discriminant functions describing shape variation in male genitalia and the first three discriminant functions describing shape variation of female genitalia. This resulted in a total of nine correlations. We corrected for the use of multiple tests using the false discovery rate in the LBE 1.22 software package in R (Dalmasso et al. 2005; R Development Core Team 2014). The presence of significant correlations between line means of the discriminant functions describing among line variation in male and female genital shape is consistent with evidence for correlated evolution of these traits.

## Results

### DOES SELECTION ON REPEATED MATING RATE LEAD TO CHANGES IN THE SHAPE OF MALE GENITALIA?

**Figure 2:**
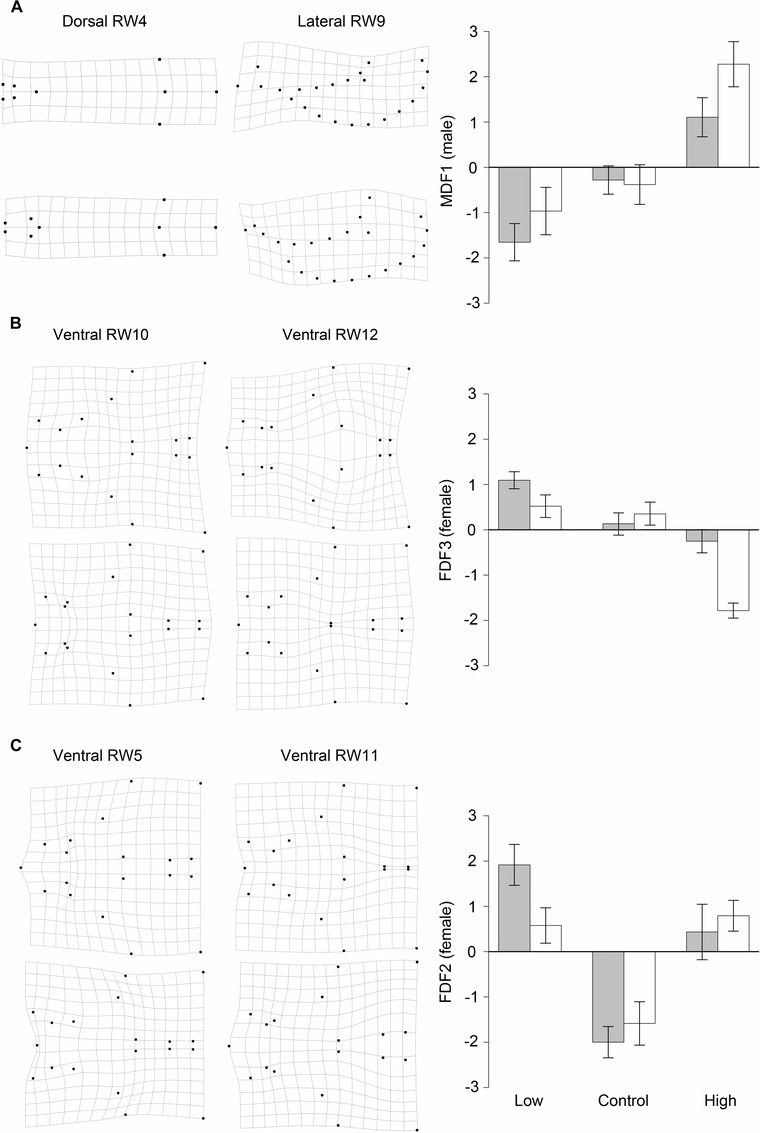
Morphological responses among lines selected for mating rate in (A). Male setae length, and paramere extension relative to median lobe; (B). Female width of vulval claws and claw extension relative to the vulva; (C). Female vulval claw shape relative to the length of the vulva. Bar charts (right) show selection line means (±CI) of discriminant functions. Solid gray bars denote the first replicate and open bars the second replicate of each treatment. Extreme positive (top left) and negative (bottom left) values of relative warps comprising discriminant functions are graphically represented by thin‐plate splines, that is dorsal relative warp 4 and lateral relative warp 9 (MDF1, males); ventral relative warps 10 and 12 (FDF3, females) and ventral relative warps 5 and 11 (FDF2, females).

The canonical discriminant function analysis identified five axes of shape variation in male genitalia. The first axis (MDF1) explained 38.8% of male genital shape variation between selection lines, and describes variation in how far the parameres extend past the median lobe, length of the terminal paramere setae (dorsal relative warp 4, Fig. 2A) as well as curvature of the parameres (lateral relative warp 9, Fig. 2A). Individuals with high MDF1 scores had long straight parameres with short setae. The second axis (MDF2) explained 28.2% of male genital shape variation between selection lines and describes variation in the distance between the terminal tips of the parameres (i.e., their “openness,” dorsal relative warp 1) and the curvature of the overall structure including parameres and phallobase (lateral relative warp 2). Individuals with high MDF2 scores had highly curved structures with widely set parameres. The third axis (MDF3) explained 17.6% of male genital shape variation between selection lines and describes variation in the relative positioning of the terminal ends of the parameres and the terminal ends of the setae (dorsal relative warp 6) as well as curvature of the whole structure (lateral relative warp 2). Individuals with high MDF3 scores had narrowly set parameres with outwardly pointing setae and low curvature of the parameres and phallobase. The remaining two discriminant functions each explained less than 10% of the variation in genital shape and so are not considered further. Relative warps and how they contribute to each discriminant function are given in the supporting information (Table S2.1).

Of these three discriminant functions MDF1 differed among selection regimes: selection on high and low repeated mating rate caused divergent evolution of male genital shape with males from lines selected for high repeated mating rates having shorter setae and parameres that extended further past the median lobe than control lines, while males from lines selected for low repeated mating rate had longer setae and parameres that did not extend as far past the median lobe than control lines (F_2,2.998_ = 15.151, *P* = 0.027, Fig. 2A). MDF2 and MDF3 did not differ among selection regimes (MDF2 – F_2,3.001_ = 2.990, *P* = 0.193; MDF3 – F_2,2.998_ = 0.126, *P* = 0.886).

### DOES SELECTION ON REPEATED MATING RATE LEAD TO CHANGES IN THE SHAPE OF FEMALE GENITALIA?

The canonical discriminant function analysis identified five axes of shape variation in female genitalia. The first axis (FDF1) explained 45.0% of female genital shape variation between selection lines, and describes the width of the vulval opening, width of the base (ventral relative warp 4) as well as the extension of the base collar up the vulval claw and the extension of the proctiger past the vulval lobes (ventral relative warp 2). Individuals with high FDF1 scores had wider vulval openings, wider bases, greater proctiger, and collar extension. The second axis (FDF2) explained 27.5% of female genital shape variation between selection lines and describes variation in the shape of the vulval claw (ventral relative warp 11 and 5, Fig. 2C) and the length of the vulva (ventral relative warp 5, Fig. 2C). Individuals with high FDF2 scores had short vulvas and shorter thicker claws. The third axis (FDF3) explained 11.7% of female genital shape variation between selection lines and describes variation in how far the vulval claws extend up the vulva (ventral relative warp 10, Fig. 2B) and the openness of the claw base (ventral relative warp 12, Fig. 2B). Individuals that had high values of FDF3 had narrow‐set claws that extend further up the vulva. The remaining two discriminant functions each explained less than 10% of the variation in genital shape and so are not considered further. Relative warps and how they contribute to each discriminant function are given in the supporting information (Table S2.2).

Of these three discriminant functions FDF2 was statistically significantly different among selection regimes: selection on both high and low repeated mating rate led to female genitals having shorter vulvas and shorter thicker claws than females from control lines (F_2,2.948_ = 15.117, *P* = 0.028, Fig. 2C.). FDF1 and FDF3 were not significantly different among selection regimes (FDF1 – F_2,3.002_ = 0.027, *P* = 0.974; FDF3 – F_2,3.007_ = 3.841, *P* = 0.149).

### ARE CHANGES IN GENITAL SHAPE OF MALES AND FEMALES CORRELATED?

**Figure 3:**
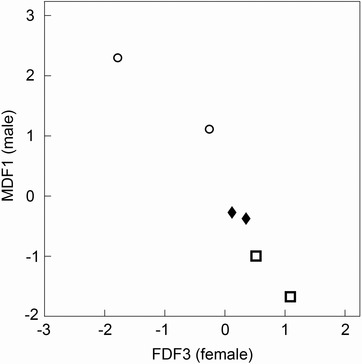
Coevolution of male and female genital shape. Plot shows relationship between male discriminant function 1 (MDF1, *y* axis), and female discriminant function 3 (FDF3, *x* axis). Open circles = lines selected for high repeated mating rate; open squares = lines selected for low repeated mating rate; solid diamonds = controls. Shape differences for the relative warps that the discriminant functions represent on this figure are shown in Figure 2A and B.

Of the nine tests examining the relationship between line variation in male genital shape and line variation in female genital shape only MDF1 and FDF3 showed a statistically significant correlation (*r* = –0.965, *P* = 0.002, Fig. 3), which remained statistically significant after controlling for multiple tests (p_FDR_ = 0.018). This relationship shows that selection lines that evolve to have males with long straight parameres and short setae also evolve to have females that have narrow‐set claws that extend further up (alongside) the vulva. Both male and female genital shape along these axes have diverged from the control lines with the divergence significant for males but not for females (see above).

## Discussion

Genital morphology evolved in *N. vespilloides* when we selected for high and low repeated mating rate, and this evolution was rapid given both male and female genital morphology evolved after only 10 generations of selection. This evolution occurred under enforced monogamy that removed the potential for mate choice and male–male competition. Males in lines selected for high repeated mating rates had shorter setae, and parameres that extended further past the median lobe than did males in control lines, while males from low lines had longer setae, and parameres that did not extend as far past the median lobe (Fig. 2A). In both high and low lines female genitals had shorter vulvas and shorter thicker claws than those of females in control lines (Fig. 2C). Male and female genitals coevolved among selection lines: lines with males that evolved long straight parameres and short setae had females that evolved narrow‐set claws extending further up (alongside) the vulva.

Thus far the best support for a significant role of sexual conflict in the coevolution of genital morphology comes from recent studies of guppies, *Poecilia reticulata* (Evans et al. 2011; Evans et al. 2013) and comparative studies of seed beetles. (Rönn et al. 2007), and water striders (Arnqvist and Rowe 2002; Perry and Rowe 2012). In leiobunine harvestmen (Opiliones) the coevolution of male and female genital structures appears to be influenced by eco‐evolutionary feedbacks related to resource availability (Burns and Shultz 2015). These studies provide strong support for the role of sexually antagonistic coevolution in producing patterns of genital divergence across species and populations but also highlight the potential dynamic relationships among the mechanisms of selection responsible. Here, we showed that directly manipulating a known mating conflict trait leads to rapid genital coevolution. The selection regime used here produces lines in which resolution of conflict between males and females is biased toward one sex or the other. The conclusion follows that there are likely to be functional correlations associated with the axes of evolved genital morphological structures that are important in controlling mating rates and maintaining a “balance of power” between the sexes. This possibility could potentially be examined in the future by reversing the direction of selection within lines with the prediction that the change in genital morphology would also be reversed. Although it is beyond the scope of the present study on its own to identify the specific mechanisms of selection that led to this pattern (e.g., we cannot categorically dismiss the possibility that we may have exposed a genetic linkage whose origin lies in cryptic female choice or elsewhere) our results strongly suggest that genital morphology can respond to selection that influences the resolution of sexual antagonism. Combining the phylogenetic approach of Burns and Shultz (2015) with our approach may be a powerful way of resolving interactions between mechanisms of selection.

There is still a puzzle in that the direction of the female response to selection in (FDF2) was the same in both high and low lines (Fig. 2C). One possibility is that the female response seen in this study is a correlated response to male genital evolution. If this were the case the direction of the response is expected to be more predictable in males, and also stronger, than that in females. For example, in a recent study that directly tested the evolutionary response in male and female genitalia to changes in sexual conflict, Cayetano et al. (2011) found that while male genitalia evolved rapidly and predictably, female genitalia did not respond. Our results, show a relatively weak response in female morphology compared to males and also apparent differences between males and females in the extent of divergence from control lines along the correlated axes (i.e., divergence was stronger for males than in females). This is broadly consistent with the view that female genital morphology evolved as a result of intersexual genetic correlation or even genetic hitchhiking. However, this view does not provide a complete picture. Because male and female genitals differ it is difficult to evaluate functional significance based on the extent of divergence in each sex. Moreover, evolution of female genital traits may be subject to constraints due to multiple functions (e.g., egg laying), which may limit the ability of females to respond to selection on male traits.

The pattern of divergence in the correlated axes of at least some aspects of male and female genital shape followed the direction of artificial selection on repeated mating rate, with high lines at one end of the relationship, low lines at the other and controls in between (Fig. 3). The magnitude of genital divergence among selection lines mirrors the response of repeated mating rate with high lines diverging further from control lines than low lines (see Fig. S3, and Carter et al. 2015 supporting information). This, and the striking mirror image of the male and female correlated response (i.e., Fig. 2A and B) indicates that the sexes have responded one to the other. We argue that this supports sexually antagonistic coevolution because of the difference in sexual conflict in our lines and because our experimental selection regime limited the opportunity for inter‐ and intrasexual selection, and thus cryptic female choice. In *N. vespilloides*, repeated mating provides direct fitness benefits for males (Bartlett 1988; Müller and Eggert 1989; Müller et al. 2007). However, an increase in mating rate apparently reduces maternal care, leading to fecundity costs to females both when increased mating frequency is the result of artificial selection (Head et al. 2014) and when females are mated more as a result of males responding to increased threats to their paternity (Hopwood et al. 2015). Repeated mating rate appears to be primarily under male control leading to the evolution of “persistent males” and “resistant females” under sexually antagonistic coevolution (Head et al. 2014).

We observed female behavioral resistance consisting of wrestling, kicking, and curling the abdomen away from the male (see also Head et al. 2014) but the measure of repeated mating on which we based selection was successful copulations. Females in nature might employ selective resistance to hinder penetration by nonpreferred males (Blanckenhorn et al. 2000; Eberhard 2002) theoretically limiting direct costs from excessive mating while still gaining indirect benefits from a successfully coercive male (Kokko et al. 2003; Kokko 2005). Commonly observed resistance behaviors in insects such as running away or kicking can be generally effective against a suite of different male genital adaptations and thus shared across taxa (e.g., Crudgington and Siva‐Jothy 2000; Blanckenhorn et al. 2002; Perry et al. 2009). Longer parameres might facilitate successful insertion and anchorage of male genitalia perhaps affecting mating rate when males struggle against female resistance. The relationship between genital structures and how they affect mating rate and/or mating success is not known at present but may be testable in future experiments (e.g., Hotzy et al. 2012; Dougherty et al. 2015).

Because we eliminated female choice and sperm competition, coevolution could have occurred because genital morphology shares a similar developmental basis in both sexes. Increased mating rate can in itself be costly to females independent of the phenotype of the male (e.g., Priest et al. 2008). In such cases genital morphology could be selectively neutral in either one sex or the other (e.g., females that employ behavioral resistance against male genital adaptations or males that increase mating rate against female genital adaptations) with genital coevolution driven indirectly in the other sex through pleiotropy. Nevertheless, our selection lines still represent the pattern of a “high line” male advantage and “low line” female advantage.

## Conclusions

Our experimental evidence suggests that sexual conflict can result in the rapid coevolution of male and female genitalia. Genital morphology of lines selected for high and low repeated mating rate diverged from controls after 10 generations of selection. The greatest divergence in morphology corresponded with lines in which the resolution of sexual conflict over mating rate was biased in favor of male interests. Future studies are needed to further understand the relative influences of different mechanisms of selection by including the eco‐evolutionary context and functional payoffs associated with genital morphological adaptations. Achieving these goals will be an important next step toward better understanding of the selective processes underlying the maintenance of sexually dimorphic traits in general.

Associate Editor: A. Maklakov

Handling Editor: M. Servedio

## Supporting information

Additional Supporting Information may be found in the online version of this article at the publisher's website:

**S1**. Supplementary data analysis and results.
**Table S1.1**: Effects of selection regime on male genital shape variation.
**Table S1.2**: Effects of selection regime on female genital shape variation.
**Table S2.1**: Relative warps obtained from the geometric morphometric analysis of male genitalia.
**Table S2.2**: Relative warps obtained from the geometric morphometric analysis of female genitalia.
**Figure S3**: Absolute response of lines selected for high mating rate (H1 & H2); low mating rate (L1 & L2) and controls (C1 & C2).
