# Supplementary material for: Selection on an antagonistic behavioral trait can drive rapid genital coevolution in the burying beetle, Nicrophorus vespilloides
Source: Evolution. 2016 May 24;70(6):1180–8. doi: 10.1111/evo.12938 (PMC5089618; doi:10.1111/evo.12938)
Supplement: Supplementary file 1 — S1. Supplementary data analysis and results. Table S1.1: Effects of selection regime on male genital shape variation. Table S1.2: Effects of selection regime on female genital shape variation. Table S2.1: Relative warps obtained from the geometric morphometric analysis of male genitalia. Table S2.2: Relative warps obtained from the geometric morphometric analysis of female genitalia. Figure S3: Absolute response of lines selected for high mating rate (H1 & H2); low mating rate (L1 & L2) and controls (C1 & C2). [file EVO-70-1180-s001.docx]

**Supplementary material for:**

**Selection on an antagonistic behavioral trait can drive rapid genital coevolution in the burying beetle, *Nicrophorus vespilloides*.**

Paul E. Hopwood, Megan L. Head, Eleanor J. Jordan, Mauricio J. Carter, Emma Davey, Allen J. Moore & Nick J. Royle.

**S1. Supplementary data analysis and results**

In addition to the univariate nested ANOVAs presented in the main text of our manuscript we also conducted nested multivariate analyses of the effects of selection regime on the 5 discriminant functions describing male and female genital shape variation between selection lines. To do this we used generalised linear mixed models and Bayesian inference as implemented by the MCMCglmm package 2.2.12 (Hadfield 2010). We ran separate models for males and females, and these were specified as follows: Selection regime was specified as a fixed effect and selection line was specified as a random effect. Each of the 5 discriminant functions was specified as response variables. We used a Gaussian distribution. For each model we ran Markov chains for 13000 iterations with a burn in of 3000 and a thinning interval of 10. Each model used unstructured variances. We used a model with an uninformative prior, which means our models were fitted with very little a priori information about the expected parameter estimates. Deviance information criteria (DIC) were used to detect the statistical significance of fixed effect of each model. The statistical significance of the genetic estimates (i.e. how different they were from zero) was assessed using 95 % confidence intervals (CI), which were calculated from the posterior distributions using the highest-posterior-density function (HPD interval, library coda; ([Hadfield 2010](#_ENREF_1)))

**Supplementary tables:**

**Table S1.1:** Effects of selection regime on male genital shape variation. Posterior means and 95% confidence intervals obtained from MCMCglmm with selection line specified as a random nested effect. Posterior DIC comparison with the model versus the restricted fixed effect model was significant (Fixed effect: 1402.143 – only residual 1415.905).

|  | Posterior mean  (lower and upper 95%CI) | | |
| --- | --- | --- | --- |
| Discriminant function | Low | Control | High |
| MDF1 | -0.777  (-1.334, -0.277) | 0.581  (-0.196, 1.318) | 1.819  (1.066, 2.599) |
| MDF2 | 0.545  (-0.337, 1.432) | -1.408  (-2.645, -0.299) | -0.282  (-1.477, 0.764) |
| MDF3 | -0.362  (-1.504, 0.745) | 0.452  (-1.280, 1.964) | 0.498  (-1.009, 2.250) |
| MDF4 | 0.175  (-0.772, 0.978) | -0.323  (-1.438, 0.938) | -0.189  (-1.451, 1.031) |
| MDF5 | 0.158  (-0.598, 0.937) | -0.521  (-1.585, 0.482) | 0.049  (-0.942, 1.296) |

**Table S1.2:** Effects of selection regime on female genital shape variation. Posterior means and 95% confidence intervals obtained from MCMCglmm with selection line specified as a random nested effect. Posterior DIC comparison with the model versus the restricted fixed effect model was significant (fixed effect: 1240.981 – only residual 1241.278).

|  | Posterior mean  (lower and upper 95%CI) | | |
| --- | --- | --- | --- |
| Discriminant function | Low | Control | High |
| FDF1 | 0.000  (-2.525, 2.050) | 0.066  (-1.503, 1.700) | -0.233  (-2.482, 2.257) |
| FDF2 | 1.770  (0.976, 2.524) | -1.030  (-1.600, -0.485) | 1.384  (0.654, 2.261) |
| FDF3 | 0.436  (-0.761, 1.499) | 0.189  (-0.661, 1.030) | -0.933  (-2.136, 0.103) |
| FDF4 | 0.369  (-1.082, 1.991) | 0.043  (-1.049, 1.195) | -0.497  (-2.040, 1.144) |
| FDF5 | 0.420  (-0.771, 1.824) | -0.220  (-1.108, 0.749) | 0.327  (-1.180, 1.658) |

**Table S2.1:** Relative warps obtained from the geometric morphometric analysis of male genitalia. Showing the % of shape variation explained by each of these warps (only relative warps that explained up to 99% of total variance are shown) and how they load on discriminant functions.

| View | Relative warp | % of variation explained | Discriminant functions | | | | | |
| --- | --- | --- | --- | --- | --- | --- | --- | --- |
|  |  |  | MDF1 | MDF2 | MDF3 | MDF4 | MDF5 |  |
| % variation explained |  |  | 38.8% | 28.2% | 17.6% | 8.0% | 7.4% |  |
| Lateral | 1 | 53.595 | 0.097 | 0.105 | -0.465 | 0.187 | 0.073 |  |
|  | 2 | 15.653 | 0.073 | 0.633 | -0.620 | -0.383 | -0.110 |  |
|  | 3 | 8.54 | 0.139 | -0.131 | -0.060 | 0.253 | -0.006 |  |
|  | 4 | 5.932 | 0.103 | -0.024 | -0.006 | -0.170 | -0.325 |  |
|  | 5 | 3.843 | -0.145 | -0.107 | 0.497 | 0.280 | 0.177 |  |
|  | 6 | 2.746 | 0.445 | -0.054 | 0.065 | 0.168 | 0.233 |  |
|  | 7 | 2.166 | -0.156 | -0.072 | -0.302 | 0.088 | 0.245 |  |
|  | 8 | 1.698 | -0.268 | 0.346 | 0.101 | -0.186 | 0.309 |  |
|  | 9 | 1.373 | 0.812 | 0.265 | 0.238 | 0.160 | 0.293 |  |
|  | 10 | 1.143 | 0-.023 | -0.166 | 0.145 | -0.222 | 0.395 |  |
|  | 11 | 0.792 | 0.321 | 0.048 | 0.373 | -0.105 | -0.319 |  |
|  | 12 | 0.55 | -0.069 | 0.494 | 0.262 | 0.150 | 0.155 |  |
|  | 13 | 0.397 | 0.100 | -0.233 | 0.112 | -0.102 | 0.096 |  |
|  | 14 | 0.334 | -0.102 | -0.304 | 0.052 | 0.232 | -0.537 |  |
|  | 15 | 0.279 | -0.145 | -0.065 | -0.199 | -0.002 | 0.329 |  |
| Dorsal | 1 | 50.588 | -.449 | .669 | .334 | -.018 | -.063 |  |
|  | 2 | 20.813 | -.095 | -.090 | .312 | -.379 | -.192 |  |
|  | 3 | 12.038 | -.511 | .259 | -.100 | .533 | .019 |  |
|  | 4 | 5.654 | .807 | .338 | .230 | .275 | .056 |  |
|  | 5 | 4.213 | -.315 | .424 | -.323 | .089 | .250 |  |
|  | 6 | 3.53 | -.102 | -.082 | .507 | -.054 | .122 |  |
|  | 7 | 3.164 | .274 | -.373 | -.125 | -.214 | .261 |  |

**Table S2.2:** Relative warps obtained from the geometric morphometric analysis of female genitalia. Showing the % of shape variation explained by each of these warps (only relative warps that explained up to 99% of total variance are shown) and how they load on discriminant functions.

| View | Relative warp | % of variation explained | Discriminant functions | | | | | |
| --- | --- | --- | --- | --- | --- | --- | --- | --- |
|  |  |  | FDF1 | FDF2 | FDF3 | FDF4 | FDF5 |  |
| % variation explained |  |  | 45.0% | 27.5% | 11.7% | 9.9% | 5.9% |  |
| Ventral | 1 | 38.912 | -.306 | .514 | -.064 | -.150 | .248 |  |
|  | 2 | 14.385 | -.489 | -.005 | -.058 | .130 | -.165 |  |
|  | 3 | 11.144 | .221 | .514 | .194 | .229 | -.140 |  |
|  | 4 | 9.04 | .866 | -.183 | -.033 | .248 | .263 |  |
|  | 5 | 5.556 | -.177 | .608 | .444 | -.263 | -.020 |  |
|  | 6 | 5.147 | .183 | .335 | .311 | .225 | -.133 |  |
|  | 7 | 3.524 | -.371 | .554 | -.046 | -.234 | .018 |  |
|  | 8 | 2.759 | .398 | .257 | .296 | -.115 | -.082 |  |
|  | 9 | 1.971 | .377 | .256 | -.213 | -.061 | .116 |  |
|  | 10 | 1.767 | .041 | .392 | -.624 | .017 | .365 |  |
|  | 11 | 1.635 | .276 | .635 | .152 | .113 | .082 |  |
|  | 12 | 1.402 | .377 | -.383 | .461 | .228 | .216 |  |
|  | 13 | 0.837 | .069 | -.141 | .104 | .138 | -.005 |  |
|  | 14 | 0.775 | .039 | .020 | .158 | -.154 | .109 |  |
|  | 15 | 0.674 | -.482 | -.053 | .036 | -.045 | .280 |  |
| Dorsal | 1 | 25.829 | -.362 | -.072 | .306 | .188 | .009 |  |
|  | 2 | 22.063 | -.021 | -.032 | -.141 | -.145 | .480 |  |
|  | 3 | 13.837 | .299 | .033 | .226 | -.227 | -.145 |  |
|  | 4 | 12.782 | .067 | -.250 | .450 | -.681 | .317 |  |
|  | 5 | 7.888 | -.469 | .002 | -.086 | .076 | -.065 |  |
|  | 6 | 6.788 | .284 | .248 | -.030 | .058 | .381 |  |
|  | 7 | 3.312 | -.118 | -.322 | -.058 | .081 | .145 |  |
|  | 8 | 2.404 | .006 | -.119 | .083 | .204 | .212 |  |
|  | 9 | 1.432 | .084 | -.129 | .093 | .054 | .352 |  |
|  | 10 | 1.125 | .172 | .335 | -.359 | -.413 | -.354 |  |
|  | 11 | 0.926 | -.185 | -.183 | -.024 | -.080 | -.082 |  |
|  | 12 | 0.617 | -.289 | .037 | -.146 | .663 | .117 |  |

**Figure S3:** Absolute response of lines selected for high mating rate (H1 & H2); low mating rate (L1 & L2) and controls (C1 & C2).

**Reference:**

Hadfield, J. D. 2010. MCMC methods for multi-response generalized linear mixed models: the MCMCglmm R package. J. Stat. Softw. 33:1-22.
